# Improving dietary intake during lunch through the provision of a healthy school lunch at Dutch primary schools: design of a pretest-posttest effectiveness study

**DOI:** 10.1186/s12889-020-08807-1

**Published:** 2020-05-12

**Authors:** Ellen van Kleef, Frédérique C. Rongen, Monique H. Vingerhoeds, S. Coosje Dijkstra, Jaap C. Seidell

**Affiliations:** 1 Wageningen University & Research, Marketing and Consumer Behaviour group, Hollandseweg 1, 6706 KN Wageningen, the Netherlands; 2 Vrije Universiteit Amsterdam, Faculty of Science, Amsterdam Public Health research institute, De Boelelaan 1085, 1081 HV Amsterdam, the Netherlands; 3 Food, Health & Consumer Research, Wageningen Food & Biobased Research, P.O. Box 17, 6700 AA Wageningen, The Netherlands

**Keywords:** School lunch, Vegetables, Primary school, School-based intervention

## Abstract

**Background:**

Since there is a shift from eating lunch at home to eating lunch at primary schools in the Netherlands, providing a school lunch may be an important opportunity to improve the diet quality of Dutch children. Therefore, the aim of this Healthy School Lunch project is to encourage healthy eating behavior of children at primary schools by offering a healthy school lunch, based on the guidelines for a healthy diet. In this study, two research questions will be addressed. The first research question is: What and how much do children consume from a self-served school lunch and how do they evaluate the lunch? The second research question is: Do children compensate healthier school lunches by eating less healthy outside school hours? The purpose of this paper is to report the rationale and study design of this study.

**Methods:**

In the Healthy School Lunch project children in grades 5–8 (aged 8–12 years) of three primary schools in the Netherlands will receive a healthy school lunch for a 6-month period. To answer research question 1, lunch consumption data will be collected at baseline and again at 3- and 6-months. This will be measured with lunch photos and questionnaires among children. To answer the second research question, a quasi-experimental, pre-test post-test intervention-comparison group design (3 intervention schools and 3 comparison schools) will be carried out. Potential compensation effects will be measured with a single brief questionnaire among parents at the three intervention and three comparison schools at month 6 of the lunch period. The school lunch will also be evaluated by parents (discussion groups) and teachers and support staff (brief questionnaires).

**Discussion:**

Results of this study will provide valuable information to influence future school lunch interventions and policies.

**Trial registration:**

This study is registered at the Netherlands trial register (NTR): trialregister.nl, Trial NL7402 (NTR7618), registered retrospectively at 2018-11-13.

## Background

Encouraging children to eat healthy is a high priority in public health globally. This is of crucial importance so that children maintain a healthy body weight and stay physically fit. Children with a healthy body weight have a greater chance of staying that way during adulthood. Moreover, a healthy body weight reduces the risk of premature onset of illnesses later in life, including diabetes type 2 and heart diseases [1, 2].

The most important contributor to a healthy body weight is an appropriate dietary intake. Children living in the Netherlands consume a dietary pattern high in foods and beverages with high levels of sugar, salt and saturated fat such as sugar-sweetened beverages, fried foods and sweet snacks [3]. Dutch children have one of the highest intakes of sugar-sweetened beverages in Europe [4]. Especially this high intake of sugar-sweetened beverages is concerning because this is related to overweight and obesity [5]. In the Netherlands, there were 13.1% of 4–11 year old children with overweight, and 3.3% with obesity in 2017 [6]. Furthermore, the diet of Dutch children is relatively low in healthy foods like fruit, vegetables, fish and whole grain products [7]. For example, 4–12 year old children eat on average 74 g of vegetables per day while the recommended daily intake is 125 to 175 g per day or more. Ninety-five percent of the children in this age group eat less than the daily recommended amount of vegetables [7]. Specifically children with a low social economic position (SEP) are likely to consume more energy-dense foods and beverages high in sugar, salt and saturated fat compared to children from middle- and high SEP groups [8]. This was confirmed by a recent study that concluded that having a lower SEP was independently negatively associated with healthier diet among 5-year old children living in the Netherlands [9].

Almost all children spend a substantial part of their time at school, making it a suitable place for health promotion [10]. In the Netherlands, they consume part of their daily energy intake during this time by eating their lunch and morning snacks at school. We recently investigated the content and quality of packed lunches of Dutch school children which they brought from home. We observed that a typical lunch of Dutch school children consists of a sandwich with a sweet or savory topping and milk or a sugar-sweetened beverage as drink. Children consumed very little fruit or vegetables during lunch. Furthermore, children who stayed at school during lunch consumed statistically significantly more sugar-sweetened beverages compared to children who ate their lunch at home [11].

Unlike countries such as Norway, Sweden, France, the UK or the USA, there is no practice of lunch school meal programs in the Netherlands [12]. Dutch primary school children bring their own packed lunches from home or go home for lunch. However, there has been a transition towards a ‘continuous schedule’ in the last years, where children do not go home for lunch and thus eat their packed lunch at school [13]. These changes in the Netherlands create an opportunity to provide a healthy school lunch since all children eat their lunch at school. An additional advantage is that in this way children from all socio-economic backgrounds can be reached since it is known that groups with a lower SEP are usually more difficult to reach for health promotion programs. For these reasons, the school setting provides a good opportunity to reduce socioeconomic inequalities in the diets of children. This was confirmed by results from a previous randomized controlled trial performed among Danish 8–11 year old children. Andersen et al. observed that overall dietary intake was improved at the food and nutrient levels when children’s habitual packed lunches were replaced by school meals for a three-month period. For example, the intake of vegetables increased with 16% in the intervention period compared to the control period [14].

Overall, children’s diet is an important contributor to their development and growth and overall health in later life. Eating habits that are adopted in childhood often persevere into adulthood [15] and therefore it is important to teach and stimulate healthy eating practices as early as possible. Therefore, the aim of this study is to encourage healthy eating during lunch of children in the primary school setting by offering a healthy school lunch, based on the Dutch guidelines for a healthy diet. In this study, two research questions will be addressed. The first research question is: What, how much and how healthy do children consume from a self-served school lunch and how do they evaluate the lunch? The second research question is: Do children compensate healthier school meals by eating less healthy outside school hours?

## Methods

### Study design

In the Healthy School Lunch project children in grades 5–8 (aged 8–12 years) of three primary schools in the Netherlands will receive a healthy school lunch for a 6-month period (November 2018 to April 2019). The development of this healthy school lunch is guided by a study among parents and children about desired school lunch concepts [16, 17]. Important to note is that in the Netherlands, it is very common for children to take all their foods and drinks from home (morning snack and lunch). There is no opportunity to buy food or drinks at primary school and there is no national subsidized school lunch program which offers school meals. Most primary schools in the Netherlands report to have a written food policy (e.g. on what is allowed to bring) although there is no legal obligation to do so [18].

To answer research question 1, we will collect lunch consumption data by means of pictures. All intervention group children will have to complete an initial baseline measurement in the situation where they still bring their own packed lunch. They will also have to complete a second and third follow-up questionnaire at month 3 half-way and month 6 at the end of the period in which a healthy school lunch will be provided.

To answer the second research question, a quasi-experimental, intervention-comparison group design (3 intervention schools and 3 comparison schools) will be carried out. There will be no randomization of the intervention and comparison schools, as the schools had to fully agree and decide on their participation beforehand, which required consent from their stakeholders (teachers, parents, support staff). Potential compensation effects will be measured with a single brief questionnaire among parents both at the three intervention schools and at the three comparison schools at month 6 of the lunch period. The school lunch will also be evaluated by parents (discussion groups) and teachers and support staff (brief questionnaire).

Schools do not receive funding to use for implementing the school lunch themselves. The expenses for ordering lunch products will be paid by the project fund. Parents will be informed about the study by means of a letter and informational meetings. A voluntary financial contribution for the costs of the lunch will be asked from parents, although it will not be registered whether and how much individual parents will pay. A small box will be put in each classroom in which children can put their financial contribution.

Active written parental consent will be obtained for all children before the study starts. Children also give verbal consent for making a photograph of their lunch. Parents may request at any time to withdraw their children from the study. The study has been approved by the Social Ethical Committee of Wageningen University, the Netherlands and is registered at the Netherlands trial register (NTR): www.trialregister.nl.

### Recruitment

A convenience sample of three primary schools agreed to participate in the intervention condition. Initial contact was established with more than 20 schools in different parts of the Netherlands, particularly focused on Amsterdam and Ede. Schools were contacted by members of the research team to recruit them for the study. In addition, a presentation was conducted for interested school personnel at a regional meeting for school directors. Recruitment of schools was based on pragmatic reasons. Inclusion criterion was having a continuous time schedule, where children lunch at school.

A power calculation was conducted to determine the sample size necessary to detect changes in the primary outcome variable ‘vegetable consumption’. Only 6.7% of Dutch primary school children eat some vegetables at school during lunch [11]. We calculated the required sample size with a study group incidence of 13.4% of children eating some vegetables (doubling the population incidence of 6.7%). This is a conservative estimate, as for each participating child 50 g of vegetables will be available each day. In the case of doubling the population prevalence, 134 children would be needed in the study sample to reach a required power of 0.8, when the statistical analysis will be performed at the 2-tailed alpha (0.05). This calculation is made using the sample size calculator ClinCalc [19]. To account for the clustered nature of the data (classes in three schools), the need for a proper process evaluation and potential dropouts, more participants are needed.

Schools were situated in the capital of the Netherlands Amsterdam and in two small cities, i.e. Lunteren and Vlaardingen (10.000–100.000 inhabitants). Two of the three schools are situated in a district characterized by residents with a lower SEP (Amsterdam and Vlaardingen) and one in a semi-rural town with children of parents with a relatively high SEP (Lunteren). Comparison schools were recruited to match these SEP levels. All children of grades 5 till 8 were invited to participate in the study. Children of grades 1 to 4 were not invited. This was done given budget constraints and we expected that older children would be better able to reflect on their experiences in the questionnaire. Children and parents who decided to join the lunch after the start will also be enrolled. Consequently, data at baseline can be missing for these children. Letters will be sent to parents to explain the study and school lunch. At each school, information meetings will be set up to inform all parents and children. All parents of children that participate will be send a letter after about three months with an invitation to give feedback about the school lunch. This evaluation is not part of the measures, but will be used to adapt and improve the intervention if necessary.

### Dietary intervention

The dietary intervention consist of an ad-libitum self-served school lunch in the intervention schools during a 6-month period. The school lunch will be developed to be nutritionally balanced and tasty.

About 80% of all available options per week will be healthy choices according to the Dutch Nutritional Guidelines based on their levels of saturated and trans fat, added sugar, salt, dietary fibre, and energy density [20]. The daily basis of the lunch is whole-grain bread slices with sandwich fillings. Every day, raw (snack) vegetables will be provided (50 g per child per day). In addition, there will be a special lunch item, such as a vegetable soup, fruit or a boiled egg every day. In two of the three schools, there will be no lunch at Wednesday as children go home early in the afternoon.

Two lunch menus are developed for three weeks that will be repeated in a three month cycle. A dietician and nutritionist will be involved to create the menu. The menus will be inspired by a qualitative study among children, parents and school staff [21], experts and chefs who provided input during workshops to make sure that the lunch options are healthy, varied and tasty. Religious or allergy-related restrictions will be taken into account. Teachers or support staff will be trained to order foods each week in a specially developed ordering system. In consultation with the school it will be determined how the lunch is organized logistically and practically. This is possible, for example, with volunteers such as parents or support staff who are already working at school. Members of the research team will visit schools regularly to provide support on this process. In case a product will be disliked by a large number of children even after repeated exposure, it will be replaced by another product. This will be decided based on these school visits by members of the research team.

Children consume the lunch in the classroom, which is similar to where they eat their current packed lunch from home. During the intervention, all children receive a cup and a lunch box that can be used as a plate to eat from during lunch. Children bring this lunch box and cup home to be cleaned and bring it back to school the next day. Equipment such as a fridge and serving plates to be put in school or classroom will be bought by the project team if needed. Children will be able to select lunch with food of high nutritional value by walking along a buffet at which all options are displayed. The buffets are displayed in the class room (one school) or in a central place in the school (two schools). Teachers are free to organize distribution in different ways, for example by putting products at groups of desks. Ideas and inspiration for healthy toppings on bread will be provided to the children by means of printed placemats and posters that will be displayed during lunch. Children are still allowed to bring a lunch item from home if they would like. Responsible school staff will receive instructions on how to deal with food safety by visits of the research team.

### Outcome measures

#### Consumption and evaluation of lunch by children

At baseline, lunch intake data of vegetables, bread, toppings, drinks and additional products (e.g. snacks) will be collected as primary outcome measures within one month before start of the school lunch. To assess lunch intake at school, the lunch of each child will be photographed just before and after consumption, and lunch components will be noted on an observation form. At baseline, children will be asked to open their lunch box and drink cup so that the products can be photographed. For different classes and schools this will be done at different weekdays to capture the variation in food offerings. Each product in the lunch box and drink cup will be classified on its healthiness by using the criteria of the Dutch nutrition guidelines: fit within recommended Wheel of Five, weekly choice or daily choice. We consider the food groups ‘vegetables’, ‘whole grain bread’ and ‘milk products and tea without sugar’ as healthy, given that they are included in the Wheel of Five, the Dutch national counselling model based on their association with a reduced risk for chronic diseases. Food groups which are advised to be limited are considered unhealthy, such as processed meats and sugar sweetened beverages [22]. For all the products, an estimation of consumption amounts will be made based on the pictures and an observation form [23]. No software will be used to determine portion size. Observers record the type of drink present and also provide an estimation of the portion size (i.e. about 200, 300, 500 ml), number of bread slices with topping present, margarine or butter spread. This measure will be repeated at T1 (after three months) and T2 (after six months, see also Table 1).

**Table 1:** Overview of included outcome measures (children)

Outcome measures	Time of measurement	T1 (after 3 months)	T2 (after 6 months)
	Baseline T0		
*Primary outcome measures child*			
- Lunch intake: observation by photographing lunch with questionnaire among children	X	X	X
*Secondary outcome measures child*			
- Evaluation and satiation after lunch: questionnaire among children	X	X	X

The secondary outcome measures for children will be collected by means of a questionnaire and involve their evaluation of the lunch and the post-lunch satiation they experience. The evaluation of the school lunch was added by the item ‘The lunch was tasty’ and ‘We had a good time during lunch’ (5-point scale). An item was included to assess the time to eat the lunch: ‘I had enough time to eat’. Response categories will be depicted via smiley faces. After introduction of the school lunch, the following items will be added to evaluate the school lunch ‘there is enough choice at lunch’, ‘I like it to prepare my own sandwich in class’, and ‘I prefer to bring my own sandwiches from home’.

To assess children’s evaluation of the quantity of lunch consumed, children will be asked the question ‘How much did you eat’ with three answer categories: ‘too little’, ‘sufficient’ and ‘too much’. To measure overall satiation after lunch, we use the specifically designed measurement instrument of Faith and colleagues [24] who use an ordinal silhouette scale with increasing numbers of circles in the stomach region to indicate increasing levels of fullness.

In the follow-up questionnaire, children will provide their age, gender and ethnicity. Children will complete the survey on their own in the classroom after lunch. Questionnaires will be administrated using EyeQuestion software at Lenovo tablets. A similar data collection procedure will take place after three months (T1) and six months (T2). At those time points, questions will be added about whether they have eaten the products of the lunch menu and their evaluation of these products.

#### Evaluation of school lunch and compensation effects as assessed by parents

A questionnaire will be administrated among parents of the three intervention schools and three comparison schools to assess potential compensation effects. Potential compensation effects due to the school lunch are probably small. To be able to measure these small effects on total dietary intake per day, several full measuring days would be required per child which makes it unlikely that the majority of children and parents would participate in extensive dietary intake measures (especially for control schools). If there are any compensation effects after school, then parents are probably the first to notice. That is why we have opted for a direct method of explicitly asking parents if they see differences in their child’s dietary intake. As parents of intervention schools are aware of the objective of the healthy school lunch, they could be more inclined to give socially desirable answers. Therefore, it is important to compare their answers with parents from schools without intervention.

To better understand how parents evaluate the school lunch and perceive their children’s eating and drinking during school lunch, one group discussion with parents at each of the intervention schools will be conducted.

##### Brief questionnaire on potential compensation effects

This questionnaire focuses on parents’ perception of changes in consumption outside school hours during the final month of the school lunch period. To increase the response rate, the questionnaire was kept very brief and were told to keep their oldest child at primary school in mind. Questionnaires will be distributed both via school e-mail and paper.

First, parents will be told the following: ‘We are curious to know whether your child started to eat differently during the past six months (since autumn holiday till now). Could you indicate whether your child eats less, about the same or more of the following products at home compared to six months ago? If you do not know, then you fill in ‘I do not know’. The following products were then mentioned: ‘snack vegetables, such as tomatoes, cucumber, radish, carrot’, ‘vegetables during the evening meal, warm or cold such as lettuce and salad’, ‘sweet snacks such as cookies, chocolate or candy’, ‘savory snacks, such as crisps, cheese or warm snacks’, ‘juice, lemonade or soft drinks with sugar (no diet)’ and ‘water, milk and sour milk’. Answer possibilities will be: ‘eating less’, ‘eating about the same’, ‘eating more’ and ‘I do not know’.

Next, parents will be asked ‘Does your child has now more or less appetite (desire for food) immediately after school compared to six months ago?‘ with answer options ‘now less appetite’, ‘about the same appetite’, ‘now more appetite’, and ‘I do not know’. The same question was asked regarding appetite during the evening meal.

Finally, parents were asked to indicate the group of their oldest child at the school and were given the opportunity to comment to the question: ‘Are there other things that you noticed with regard to the eating behavior of your child in the past six months? For example, are there foods or drinks your child asked for or talked about? Do you have any more suggestions or questions for the researchers?’

##### Group discussion on evaluation school lunch

At each intervention school, a group discussions will be conducted using an interview guide (Table 2). This semi-structured interview guide contains both closed- and open-ended questions. The discussions will take place at school after school hours in the afternoon and evening and will be facilitated by at least two researchers. Discussions will take about 45 min and will be audiotaped. Parents will be recruited at school on a convenience basis using the school newsletter and posters.

**Table 2:** Interview guide for parents’ group discussion

Stage	Content
1) Introduction and welcome	Informed written consent, explanation of discussion topic and rules, introduction of participants
2) Parents’ general school lunch evaluation	Individual task: Rating of three statements Group discussion of task
3) Perceptions of child’s eating and drinking during school lunch	Individual task: Rating of four statements Group discussion of task
4) Questionnaire	Individual completion of questionnaire with demographics.

First, participants will be to provide written informed consent. After a warming up round of introductions of each participant, they will asked about their general evaluation of the provided school lunch by responding individually to four statements: ‘My child is satisfied with the school lunch’, ‘I am satisfied with the school lunch’, ‘My child prefers a home-brought lunch’ and ‘I prefer a home-brought lunch’. Each statement has to be evaluated on a 3-point scale (disagree, do not know/in doubt, agree). Subsequently, the moderator will ask participants to discuss their responses to the statements plenary. Follow-up questions will be used to gain more in-depth insights.

Next, participants will be asked to indicate individually the level of disagreement or agreement to the statement ‘My child eats and drinks …. during lunch at school’, followed by ‘healthy’, ‘sufficiently’, ‘tasty’ and ‘varied’ on a 3-point scale (disagree, do not know/in doubt, agree). Answers will be discussed in the group. Participants were probed for the underlying reasons for their answer. Finally, each participant will complete a background questionnaire to determine age, gender, group and age of children and highest completed educational level of the participating parent. Participants will receive a small gift.

#### Evaluation of school lunch by teachers and support staff

Teachers and support staff’ perceptions of child’s eating and drinking behavior during school lunch will be assessed two times (T1 and T2) by a questionnaire with three-point Likert agreement scales (disagree, neutral, agree) to indicate the level of disagreement or agreement to the statements ‘Children generally like the lunch’, ‘Children have enough time to eat’, ‘Children find it cozy during lunch’, ‘There is sufficient of everything’, ‘Lunch is ready on time’, ‘Lunch is cleaned up on time’, ‘The atmosphere is good during the lunch’, and ‘The atmosphere in class is good during the afternoon’. Teachers could also indicate ‘not applicable’. These items were informed by previous research on the introduction a healthy school lunch in the Netherlands [16, 26].

### Data analysis

#### Consumption and evaluation of lunch by children

The unit of analysis will be the child. The primary outcome measures of this study is the food and drink consumption of the children during lunch, which is measured by analyzing photographs of the lunch of each child. Vegetable portion size in grams will be estimated based on the pictures and observation forms. Bread will be reported in type of bread and slices, crackers and fruit in pieces and drinks in milliliters. Of all products, intake will be assessed by subtracting the portion after from the portion before and tested for statistically significant differences. Important outcomes will be the incidence of children consuming a particular product. Success of the intervention will be indicated by at least 50% of the children consuming at least a portion of 25 g of vegetables during lunch.

The number of different products per lunch will be calculated. Next, the share of healthier ‘Wheel of Five’ products consumed will be plotted and compared across time using chi-square analyses and correlations. Acceptability of the school lunch is assessed by the questionnaire item ‘the lunch was tasty’ assessed at T1 and T2. Adequate acceptability will be indicated by 75% of the children reporting a mean rating of three or higher.

#### Evaluation of school lunch and compensation effects as assessed by parents

Descriptive statistics will be used to examine the compensation questionnaire among parents. Chi-square tests of independence will be computed for ordinal answers on compensation questions in the questionnaire to identify statistically significantly differences among parent groups (comparison versus intervention schools).

Each session of the group discussions with parents will be audiotaped. During the sessions, notes will be taken by at least two researchers. After the discussion, these two researchers will discuss and summarize their main findings.

#### Evaluation of school lunch by teachers and support staff

Descriptive statistics will be used to examine the questionnaire among teachers and support staff.

## Discussion

This study will be conducted to assess the impact of providing a healthy school lunch on dietary intake of children during lunch of primary schools in the Netherlands. The primary outcome measures of this study focus on how much and healthy children consume from the school lunch. A secondary objective is to assess the degree of compensation of eating less vegetables, or more unhealthy snacks and drinks outside school hours. Lunch at school is typically not provided in the Netherlands, but has traditionally been consumed at home or brought from home by children themselves and eaten at school. A healthy lunch provided by school therefore presents many changes for children, parents and the school. We hypothesize that the school lunch will lead to an increase in vegetable consumption.

A strength of this study design is that it focuses on the development of a school lunch that is acceptable for children, parents and schools in various ways. The lunch was tailored to their needs to improve the likelihood of acceptance and the evaluation of the school lunch will be assessed among both children, parents and school teachers. In this way, it is most likely that healthier school lunch eating behavior is encouraged. Moreover, we measure potential compensation effects of the school lunch as assessed by parents, as unwanted compensation effects are a major concern when improving school food environments [25].

Some limitations should be noted about the design. Only three intervention and three comparison schools participated with children of grades 5 till 8. Moreover, there may be other explanations of why consumption of key food groups of interest may have changed during the school day. The limited intervention period of six months may be insufficient to obtain sustainable behavior changes and acceptance of the lunch. In addition, a voluntary financial contribution will be asked from parents, but this may not be feasible for certain income groups. These issues are important to consider in future research and implementation practices.

As already experienced in the recruitment of intervention and comparison schools, commitment is essential to effectively implement the intervention. A major bottleneck for participation of schools is the constraints they feel in lack of time and resources [26]. Nevertheless, we were able to recruit schools based on motivation, which reflects the real-life situation of many public health interventions which are not legally required. This is believed to facilitate implementation in the future [21]. By building on a thorough assessments of needs and preferences among the target group, the intervention was carefully constructed to fit the needs of all involved. As such, the intervention is contextually developed to increase the likelihood that this school lunch is scalable to other schools in the Netherlands. Feasibility issues will be very important in this respect and therefore a process evaluation will be carried out and reported separately.

Results of this study will provide valuable information for designing primary school based meal intervention studies and policies and if successful help to improve the health of school children.

### Study status

At the time of the first submission, the intervention and data collection was completed in April 2019. Data is currently being analyzed.

## Abbreviations

SEP: Social Economic Position
WHO: World Health Organization
CBS: Centraal Bureau Statistiek
